# Early Outcomes of State Public Health Actions’ School Nutrition Strategies

**DOI:** 10.5888/pcd14.170106

**Published:** 2017-12-07

**Authors:** Seraphine Pitt Barnes, Syreeta Skelton-Wilson, Adina Cooper, Caitlin Merlo, Sarah Lee

**Affiliations:** 1 Centers for Disease Control and Prevention, Atlanta, Georgia; 2 ICF Macro, Atlanta, Georgia

## Abstract

**Introduction:**

Since 2013, the State Public Health Actions to Prevent and Control Diabetes, Heart Disease, Obesity and Associated Risk Factors and Promote School Health (State Public Health Actions) program has been implemented to support and reinforce healthy choices and healthy behaviors among the US population. The Centers for Disease Control and Prevention’s Division of Population Health’s School Health Branch has been a critical component, ensuring that state health departments support schools in adopting nutrition standards and creating a supportive nutrition environment. The objective of this article was to describe early outcomes of the school nutrition strategies of State Public Health Actions.

**Methods:**

We examined the extent of progress for short-term performance measures and for school nutrition evaluation questions, using data secured from 51 grantees through the performance measures database and state evaluation reports.

**Results:**

During the first 4 years of the cooperative agreement, grantees demonstrated significant progress compared with year 2 for school nutrition performance measures. Collectively, grantees provided professional development and technical assistance to staff in 7,672 local education agencies and reached more than 29 million students. Success was also noted for several nutrition practices in schools.

**Conclusion:**

These early outcomes suggest that State Public Health Actions has had a positive impact on the nutrition environment of US schools. Systematically addressing areas for improvement could further expand the reach of these efforts during the remainder of the cooperative agreement.

## Introduction

State Public Health Actions to Prevent and Control Diabetes, Heart Disease, Obesity and Associated Risk Factors and Promote School Health (State Public Health Actions) is a 5-year cooperative agreement that supports state health departments in the promotion of healthy choices and healthy behaviors among the US population (1). The Centers for Disease Control and Prevention’s (CDC’s) Division of Population Health, School Health Branch seeks to prevent childhood obesity through healthy eating and physical activity policies and practices and through supporting students with chronic health conditions.

More than one-third of children and adolescents in the United States are overweight or obese (2). Because many students consume up to half of their daily calories at school (3), the school nutrition environment can affect children’s diet and overall health. Schools are a priority setting for addressing healthy eating behaviors through access to healthy foods and beverages and obtaining consistent information about healthy eating (4–6). The school nutrition environment includes foods and beverages available through multiple venues (eg, school meal programs, vending machines, celebrations, fundraisers), messages about good nutrition that students see and hear, and opportunities for students to learn about and practice healthy eating behaviors (7). Although recent policy changes at the federal, state, and local levels have improved the school nutrition environment, continued progress on policy implementation is needed (8–10).

This article describes the early outcomes of the school nutrition strategies, specifically changes in school-level nutrition practices, of State Public Health Actions.

## Methods

In June 2013, all 50 states and the District of Columbia were awarded funding under either the basic or enhanced component of State Public Health Actions (1). The basic component awarded funding to all states to support health promotion, epidemiology, surveillance activities, and targeted strategies. The competitive enhanced component awarded additional funding to 32 states to build on and extend the activities supported with basic funding to achieve greater reach and impact. In this study, we examined data from grantees that reported use of these funds to adopt nutrition standards and create supportive nutrition environments in schools. The required strategies focused on interventions that included providing professional development and technical assistance; establishing standards (including for sodium) for all competitive foods (ie, foods and beverages sold outside of the school meal programs); prohibiting advertising of unhealthy foods; and promoting healthy foods in schools, including those sold and served in school meal programs and other venues. Grantees are required to report component-specific performance measures annually and provide data on year 5 targets; states that receive enhanced funding are required to report outcomes. In addition, grantees that receive enhanced funding are expected to work with up to 15 local education agencies (LEAs) (ie, school districts). States identified LEAs, which represent a subset of all the LEAs in a state, on the basis of their own criteria; however, they were encouraged to select LEAs in areas disproportionately affected by chronic diseases and the risk factors that cause them, including those with students who have a high prevalence of overweight or obesity, have limited access to healthy foods and beverages, and do not obtain adequate physical activity. The analysis and findings presented in this article are a subset of a national evaluation (11), and approval was secured from the Office of Management and Budget (no. 0920–1059).

We conducted a document review of annual progress reports and analyzed quantitative performance measure data using a mixed-methods design. These data represented performance from July 1, 2013, through January 31, 2017. Data were obtained from 51 grantees that reported on basic performance measures; 32 of those grantees also reported on enhanced performance measures for enhanced states. School Health Profiles (Profiles) was the primary data source for all school-level performance measures. Profiles is a system of surveys that assess school health policies and practices among secondary schools in states, territories, tribal jurisdictions, and large urban school districts (12). Profiles uses random, systematic, equal-probability samples to produce data representative of schools with 1 or more of grades 6 through 12 in each site. Data were collected in spring 2014 and 2016. Profiles data were collected in the targeted LEAs identified in each of the 32 enhanced states. The primary data source for the policy-related performance measures (ie, 2.3.03 and 2.3.05) was WellSAT 2.0, an interactive tool used to assess the quality of written local school-wellness policies (13). Grantees also developed their own data collection systems to track professional development and technical assistance. Only data from years 2 and year 4 were included for all measures.

In addition, we collected and analyzed year 3 state evaluation reports. These evaluation reports represented data collection from August 1, 2015, through July 31, 2016. Grantees submit annual evaluation reports that provide detailed findings derived from their state-level evaluation of interventions and strategies. All grantees submitted evaluation reports, of which 29 of the 32 enhanced grantees evaluated the implementation of the enhanced school nutrition strategy. Evaluation reports addressed core questions corresponding to level of implementation. Grantees self-identified as being in either the adoption or implementation phase for the school nutrition strategy. Grantees at the adoption stage reported findings in response to the following core questions: 1) “What state activities have been effective in promoting nutrition policy development and nutrition practice adoption among districts and schools?” and 2) “What are the major facilitators and barriers in helping districts and schools create a supportive nutrition environment, such as partnerships with the Department of Education? How were the barriers overcome?” Grantees at the implementation stage reported findings related to the following core questions: 1) “What critical factors or activities influence the successful implementation of nutrition policy and nutrition practice?” and 2) “To what extent has implementation of nutrition policies and nutrition practices increased access to healthier foods and beverages at school?” In their evaluation report, grantees provided summaries of the approach and strategies of the intervention, the facilitators and barriers to implementation of the strategies, the evaluation indicators, and the evaluation findings.

For analysis of the annual progress reports, year 2 and year 4 data for eligible states were aggregated. Reach estimates were calculated on the basis of the performance measure type (ie, count, numerator and denominator, and percentage). Measures reported as counts were summed as totals across eligible states. Measures reported as percentages were reported as means across eligible states. In addition, for each performance measure, the percentage change in the actual values reported from year 2 to year 4 were calculated. Next, to determine whether the percentage change for each measure was significant (*P* < .05), the average percentage change (across all grantees) was compared with zero by using one-sample *t* tests. Achievements of basic component short-term measures are also described.

Primarily qualitative data were obtained from evaluation reports. We identified and summarized the key school nutrition evaluation findings for year 3. Thematic analysis was conducted to identify and classify patterns or themes across the evaluation reports in terms of the types of changes in the nutrition environment, facilitators, barriers, lessons learned, and recommendations reported. The analysis involved inductively coding the data, deductively identifying themes, visualizing the relationship between codes and themes, sorting and sifting themes to identify interrelationships, and constructing memoranda that summarized the themes. This method of analysis incorporated both the data-driven inductive approach of Boyatzis (14) and the deductive a priori template of codes approach outlined by Crabtree and Miller (15).

## Results

Data from the program’s fourth year of implementation demonstrated reach of professional development and technical assistance, progress on the implementation of various evidence-based practices, and identification of factors related to effective nutrition policy and practice. Data were provided by 51 grantees (all 50 states and the District of Columbia).

### Performance measures

Data from the basic component show that 7,672 LEAs received professional development and technical assistance on strategies to promote the adoption of nutrition standards, affecting 29.3 million students in these LEAs (Table) and representing a significant increase of 3,771 LEAs from year 2. Among the 32 enhanced grantees with targeted work in up to 15 LEAs each, 609 LEAs received professional development on creating a supportive nutrition environment in schools, affecting approximately 3.7 million students at year 4. The increase between year 2 and year 4 was significant.

**Table:** Performance Measures Data of State Public Health Actions

Performance Measure	Year 2 Actual	Year 4 Actual	Significance Test^a^ (*t*_df_; *P* Value)
**Basic component**			
B.1.01. Number of local education agencies that received professional development and technical assistance on strategies to create a healthy school nutrition environment	3,901	7,672	*t*_41_ = 2.66; .01
B.1.02. Number of students in local education agencies where staff received professional development and technical assistance on strategies to create a healthy school nutrition environment	17,600,600	29,313,953	*t*_41_ = 1.40; .17
**Enhanced component^b^^,^ c**			
2.3.01. Number of local education agencies that received professional development and technical assistance on strategies to create a healthy school nutrition environment	378	609	*t*_35_ = 2.54; .02
2.3.02. Number of students in local education agencies where staff received professional development and technical assistance on strategies to create a healthy school nutrition environment	2,862,354	3,695,833	*t*_34_ = 2.26; .03
2.3.03. Percentage of local education agencies that have adopted and implemented policies that establish standards (including sodium) for all competitive foods available during the school day	50.4	59.5	*t*_21_ = 1.62; .12
2.3.04. Percentage of schools that do not sell less healthy foods and beverages (soda pop or fruit drinks, sport drinks, baked goods, salty snacks, candy)	45.5	58.5	*t*_46_ = 4.73; <.001
2.3.05. Percentage of local education agencies that have adopted and implemented policies that prohibit all forms of advertising and promotion (eg, contests and coupons) of less nutritious foods and beverages on school property	29.1	33.5	*t*_18_ = 0.54; .60
2.3.06. Percentage of schools that prohibit all forms of advertising and promotion for candy, fast food restaurants, or soft drinks	55.1	56.9	*t*_47_ = 1.35; .18
2.3.07. Percentage of schools that price nutritious foods and beverages at a lower cost while increasing the price of less nutritious foods and beverages	12.3	13.1	*t*_46_ = 1.48; .15
2.3.08. Percentage of schools that provide information to students or families on the nutrition, caloric, and sodium content of foods available	50.4	54.7	*t*_47_ = 3.05; .004
2.3.09. Percentage of schools that place fruits and vegetables near the cafeteria cashier, where they are easy to access	79.2	80.2	*t*_47_ = 1.16; .25
2.3.10. Percentage of schools that allow students to have access to drinking water	58.6	57.9	*t*_47_ = 0.96; .34
2.3.11. Percentage of schools that offer fruits or non-fried vegetables when foods or beverages are offered at school celebrations	32.8	34.1	*t*_47_ = 1.59; .12
2.3.12. Percentage of schools that allow students to purchase fruits and vegetables from vending machines or at the school store, canteen, snack bar, or as à la carte items	19.9	14.4	*t*_45_ = −0.09; .93

a Significance was set at *P* <.05.

b Measures reported as mean percentages to represent the average percentages across eligible states.

c For duplicate measures, the values are independent. Number of schools surveyed in year 2 was 1,337; number of schools surveyed in year 4 was 1,250.

In the areas of nutrition policy and school nutrition practices among the 32 enhanced grantees, 59.5% of LEAs reported adopting and implementing policies to establish standards (including for sodium) for all competitive foods available during the school day, compared with 50.4% in year 2 (Table). Additionally, 58.5% of schools reported they do not sell less-healthy foods and beverages, a significant increase from year 2. In year 4 only 33.5% of LEAs reported adopting and implementing policies that prohibit all forms of advertising and promotion, while 56.9% of schools indicated they prohibited all forms of advertising and promotion for candy, fast food restaurants, or soft drinks. A little more than 13% of schools also reported they priced nutritious foods and beverages at a lower cost, and almost half of schools provided nutrition information to students or families, a significant increase from year 2. Eighty percent of schools increased accessibility of healthy options by placing fruits and vegetables near the cafeteria cashier. The percentage of schools that allow students to have access to drinking water decreased slightly, from 58.6% in year 2 to 57.9% in year 4 (Table). Nearly one-third of schools offered fruits and nonfried vegetables when foods are offered at school celebrations, and 14.4% of schools allowed students to purchase fruits and vegetables from vending machines, or at the school store, canteen, snack bar, or as à la carte items, a decrease from 19.9% in year 2 (Table).

### State evaluation

Fifty-one state evaluation reports containing results of interventions designed to create supportive nutrition environments were analyzed. Fifty-seven percent of grantees evaluated the strategy focused on creating healthy school nutrition environments (12 in the adoption phase and 17 in the implementation phase). More than half of the 29 grantees evaluating this strategy reported the facilitators and barriers to creating supportive nutrition environments. Among them, 67% identified strong leadership, committed staff, formal partnerships, and engaging champions for school nutrition as key facilitators. The availability of professional development and technical assistance to support staff in the development, implementation, and evaluation of school nutrition interventions also was beneficial. Collectively they provided more than 780 professional development and technical assistance opportunities; 11 grantees reached more than 4,200 state, district, and school administrators; food service staff; and teachers. Other facilitators included communicating about school nutrition, offering incentives, and disseminating resources. Forty-one percent of grantees also found these facilitators influential in the successful implementation of nutrition policy and practice. Sixty percent of grantees reported barriers to creating a supportive nutrition environment, including inadequate capacity (eg, lack of appropriate facilities, staff), negative attitudes, perception of changing traditions (ie, snacks for celebrations), and lack of buy-in and support.

Grantees also described many activities that contributed to the perceived increase in access to healthier foods and beverages. For 75% of grantees, the implementation of nutrition practices and policies to increase access to healthier foods and beverages in schools was largely accomplished by changing and adopting district-level nutrition policies and practices for competitive foods, healthy school celebrations, events, or fundraisers; food and beverage procurement; and meal preparation and service. Many of these policy changes were enacted to better align with state and national nutrition standards such as the new Smart Snack regulations, but some also aimed to improve the consistency and quality of how food is presented to students, availability of information about menu nutrition contents for parents and students, and access to healthier food options (ie, in vending machines and during meal service). Some of these policies also prohibited advertising and promotion of less nutritious foods and beverages on school property.

As a result of these policy changes, 66.7% of grantees saw practice changes implemented in schools and districts, including increased use of locally grown food (eg, via school gardens) and scratch cooking methods, thus improving the quality and nutritional value of school meals. Changes in local wellness policies also bolstered the development of school health improvement plans that targeted healthy eating habits; the consumption of fruits, vegetables, and healthy snacks; and use of evidence-based and comprehensive health education curricula that align with national and state standards to promote healthy behaviors among students in districts.

In addition, some districts and schools restricted food celebrations, marketing of unhealthy choices, and use of food and beverages as a reward. For example, one grantee reported that nutrition guidelines changed to require that healthy food options be made available at school celebrations, events, and/or fundraisers. Finally, grantees reported observing some improvements in the availability, quality, and selections of foods and beverages in food service lines and à la carte items among its targeted districts, and one grantee reported a 4% decline in the consumption of soda among high school students.

## Discussion

State Public Health Actions’ school health strategies aim to provide a comprehensive approach to adopting nutrition standards and creating a supportive nutrition environment in schools through professional development and training of school staff, adoption and implementation of nutrition policies, and implementation of various evidence-based nutrition practices. The findings from this evaluation provide both a broad perspective and more details of school nutrition work in the United States. For example, significant progress has been made in reaching school nutrition and other school health professionals through professional development and technical assistance. This progress enables new knowledge, skills, and abilities among key stakeholders who are also often the implementers of school nutrition policies and strategies.

Outcome data for most school-level nutrition performance measures were favorable. Grantees implemented practices such as placing fruits and vegetables near the cafeteria cashier, where they are easy to access, and prohibiting all forms of advertising and promotion for candy, fast-food restaurants, or soft drinks. These are just 2 examples of no-cost or low-cost strategies that schools can implement to create a supportive nutrition environment. Grantees also reported achieving significant increases in 2 evidence-based practices: not selling less-healthy foods and beverages (soda pop or fruit drinks, sport drinks, baked goods, salty snacks, candy) and providing information to students or families on the nutrition, caloric, and sodium content of foods available. Slight nonsignificant increases were observed in the percentage of local education agencies that adopted and implemented policies that prohibit all forms of advertising and promotion (eg, contests and coupons) of less-nutritious foods and beverages on school property, the percentage of schools that price nutritious foods and beverages at a lower cost while increasing the price of less nutritious foods and beverages, and the percentage of schools that offer fruits or nonfried vegetables when foods or beverages are offered at school celebrations. Surprisingly, decreases were observed in 2 evidence-based practices: allowing students to have access to drinking water and allowing students to purchase fruits and vegetables from vending machines or at the school store, canteen, snack bar, or as à la carte items. Although these decreases suggest an opportunity for improvement, these achievements collectively demonstrate the capacity of school nutrition nationwide (6).

These data also show that more than half of all targeted LEAs have adopted nutrition standards. These standards require schools and school cafeterias to offer more fruits, vegetables, whole grains, and fat-free or low-fat dairy products and limit sodium, added sugar, calories and unhealthy fat in competitive foods found in à la carte, vending machines, and other venues (16,17). The findings are promising, because the consumption of healthier foods by students may be influenced by implementing these nutrition standards that promote the availability of healthier options offered and purchased at school (18–20).

School nutrition policies, when implemented, support consistent and lasting change in schools. Although 56.9% of schools reported prohibiting all forms of advertising and promotion for candy, fast foods, or soft drinks, only 33.5% of LEAs adopted and implemented policies that prohibit all forms of advertising and promotion. This area is one that needs to be better understood. In practice, grantees are making progress in prohibiting advertising; however, ensuring that this practice is sustainable through the adoption of policy still needs to be improved.

Furthermore, federal legislation such as the Healthy Hunger Free Kids Act of 2010 has been a leverage point for State Public Health Actions grantees, including local wellness policy requirements, Smart Snacks in School, and school meal standards (8). State Public Health Actions grantees have not only supported LEAs and schools to implement these requirements but also helped them go above and beyond the requirements, as evidenced by the performance measures. For example, Smart Snacks in School standards apply only to foods and beverages sold during the school day, whereas this cooperative agreement and its related performance measure addresses all competitive foods available during the day. Through legislation, there will be continued opportunities for grantees to support LEAs and schools in limiting or prohibiting advertising of unhealthy food and beverages, as local school wellness policy requirements now require LEAs to include information about marketing and advertising in their local wellness policies and only market foods that meet Smart Snacks standards.

Finally, state evaluations have allowed for exploration of facilitators, barriers, and outcomes that enable both CDC and grantees to identify future training, technical assistance, and policy development needs among LEAs and schools. For example, from grantees’ evaluation reports, common facilitators have been identified, such as having strong leadership, committed staff, formal partnerships, and engaged champions. Other studies have found similar facilitators to those of grantees, suggesting that these elements support greater implementation of local wellness policies and environmental strategies to promote healthy food and beverages (21,22). These facilitators are important to not only identify but continue to support healthy school nutrition environments, policies, and practices. At the same time, grantees reported that barriers persist, such as negative perceptions (eg, of school meals, nutrition), lack of support and buy-in from key stakeholders (eg, administrators, parents), and lack of facilities and resources (eg, lack of free, filtered water; suitable space).

Although results are encouraging, our study has limitations. Many of the findings were derived from performance data from 32 enhanced grantees who were awarded funding on a competitive basis. These data, although representative of the LEAs selected, may not be generalizable to all LEAs in the United States because grantees’ used their own criteria (eg, high need, existing relationships) to select LEAs. In addition, we used self-reported data that were not verified or corroborated by other data or observations. Furthermore, grantees who choose to evaluate the school nutrition strategy may represent more highly motivated grantees, so data from these reports should be interpreted cautiously. Despite these limitations, the data we used were collected by using quality surveillance and data collection systems.

To date, close to 8,000 LEAs and more than 29 million students, representing 57% of US school districts and 59% of students, have been reached by State Public Health Actions (16). Our findings demonstrate that through professional development and technical assistance grantees increased their capacity to implement evidence-based nutrition policies and practices in US schools. The work of grantees will likely continue to have an effect on school nutrition in the final year, as local school wellness policy requirements will be implemented, equipment and facilities grants from the United State Department of Agriculture (USDA) will be available, and tools, resources, materials, and trainings will also be available through USDA, CDC, and other organizations. There will also be opportunities for grantees to support LEAs and schools in limiting or prohibiting advertising of unhealthy food and beverages. Through professional development, training, and local school wellness policy requirements, grantees will continue to work with LEAs to improve nutrition policy and implement evidence-based strategies that improve student nutrition and the school nutrition environment as a whole.

Finally, as grantees enter the final year of the cooperative agreement, they are required to develop impact statements. These impact statements will highlight the accomplishments of their work during the past 5 years. These impact statements can be used by grantees and CDC to garner support from funders and key decision makers for continued work in the area of school nutrition. 
